# The tapeworm’s elusive antero-posterior polarity

**DOI:** 10.1186/s12915-016-0244-7

**Published:** 2016-03-10

**Authors:** Alessandro Minelli

**Affiliations:** Department of Biology, University of Padova, Via Ugo Bassi 58 B, I 35131 Padova, Italy

## Abstract

Because of their sessile lifestyle and the lack of the sensory and feeding structures usually associated with the cephalic end, fixing the antero-posterior (AP) polarity of tapeworms is somewhat equivocal and has been a matter of century-long debates. Koziol et al. offer the first molecular evidence finally fixing the scolex as the animal’s anterior pole.

Please see related research article: Comparative analysis of Wnt expression identifies a highly conserved developmental transition in flatworms, http://dx.doi.org/10.1186/s12915-016-0233-x

## Morphological versus molecular markers of axis polarity

**Fig. 1:**
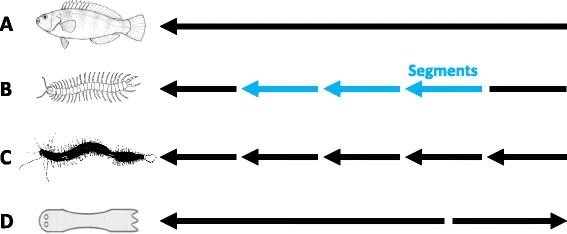
Continuity or discontinuity of antero-posterior (AP) polarity along the main body axis of different metazoans. **a** In most metazoans, the AP polarity of the main body is the same throughout the whole length of the body. **b** In arthropods, serially repeated body units (*Segments*: *blue arrows*) have the same polarity as the rest of the body but with positional values reset at each boundary between them. **c** The same pattern emerges in the case of asexual reproduction by transversal fission. However, in asexual reproduction by (posterior) budding, as in the acoel *Convolutriloba retrogemma* (**d**), the parent and the backwards-growing bud have opposite AP directional polarity

In most metazoans, it is clear which part of the body is anterior and which part is posterior. This is true even in the case of animals with serially repeated body units, such as arthropod segments and the developing zooids in animals with asexual reproduction by fission: all linearly arranged segments and zooids of the animal agree in polarity although their positional values are reset at each boundary between segments or zooids (Fig. 1a–c). Most commonly, the anterior end of the body is marked by a local concentration of nervous (brain) and sensory structures, more developed in free-living than in sessile animals, and by the position of the mouth.

**Fig. 2:**
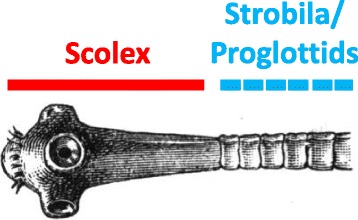
Scolex and strobila (a chain of proglottids), the structural modules of a tapeworm’s organization

Problematic, however, and a matter of dispute to this day, is the ‘body syntax’ of adult tapeworms, or cestodes. These sedentary parasitic worms, which feed by absorbing the already digested fluid food found in their hosts’ guts, do not have a mouth, or a conventional head. Their architecture includes a scolex provided with suckers and/or hooks, used to attach to the gut wall of the parasite’s vertebrate host, followed by the strobila, a chain of units (as few as four in *Echinococcus*, but up to about 4000 in *Diphyllobothrium latum* [1]) known as the proglottids (Fig. 2). Proglottids have been variously interpreted as the segments of an individual animal (for example, [2, 3]) or the zooids of an essentially colonial one (for example, [4]).

Modern zoology textbooks uniformly describe the scolex as the anterior end, but an opposite interpretation of the antero-posterior (AP) polarity of the tapeworm’s main body axis has sometimes been argued, beginning with Moniez in 1880 [5]. Debates about tapeworm polarity (for example, [1, 3, 6, 7]) have revolved on aspects of comparative morphology (the relative position of testes versus ovaries and the position of the growth zone from which the proglottids are generated) and on opposite interpretations of the architectural changes accompanying the metamorphosis of the typical oncosphere larva into postlarval and eventually adult stages.

In addition to the difficulties caused by adult morphology, the assessment of AP polarity in tapeworms is also confounded by their highly derived life cycles, which involve indirect development through very peculiar larval stages, the AP polarity of which is even more problematic, and less distinct, than the AP polarity of the adult.

Molecular markers involved in determining AP polarity are known in other animals whose body syntax is unproblematic, including planarians, free-living members of the same phylum, the flatworms, to which tapeworms belong. At last, the spatial expression patterns of these genes have been studied in two tapeworm species [8]. This study unequivocally identifies the apical part of the scolex as the anterior focus of the worm’s axial polarity, as described in zoology textbooks.

In planarians, canonical Wnt/β-catenin signaling is involved in the specification and maintenance of the AP axis. This involves gradients of specific Wnt ligands and extracellular inhibitors of Wnts expressed at the posterior and anterior body end, respectively. Koziol et al. [8] postulated that such AP polarity markers, of which there is no evidence in tapeworm larval stages, might be expressed during the metamorphosis to postlarval stages, when the worm differentiates the scolex and subsequently starts producing proglottids. This expectation proved to be correct: during larval metamorphosis there is indeed expression of posterior Wnt markers and scolex formation is preceded by localized expression of Wnt inhibitors that in planarians mark the anterior end of the body. Thus, expression of polarity markers is conserved between the early stages of larval metamorphosis in tapeworms and late embryonic and adult planarians.

## The scolex and the strobila

So, is the issue of the tapeworm’s AP polarity definitely solved? Koziol et al. [8] have clearly succeeded in fixing the polarity of the scolex, but this may not necessarily imply that the polarity of the strobila is also definitely fixed. As acknowledged by the authors, two problems remain. Granted that the scolex represents the anterior end of the animal, we would expect it to proliferate from a posterior subterminal region: if so, the oldest proglottid should be the first following the scolex and the youngest one should be the most distant from the scolex. Tapeworms, however, grow the other way: the proliferating zone remains throughout life immediately adjacent to the posterior part of the scolex, the region where Koziol et al. [8] have found the focus of Wnt expression interpreted as a posterior marker. As a consequence, the oldest proglottid is the terminal one, opposite to the scolex, and the youngest is the one closest to the scolex.

**Fig. 3:**
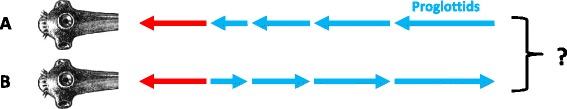
Alternative interpretations of the antero-posterior (AP) polarity of the strobila. In tapeworms, the AP polarity of the scolex (*red*) has been definitively fixed by the gene expression studies of Koziol et al. [8] in *Echinococcus multilocularis* and *Hymenolepis microstoma*. However, the verdict is still open on the question of the polarity of the proglottids. These are generally interpreted as being polarized in concordance with the scolex (**a**), but the localization of the body region from which the proglottids proliferate as well as the relative position of testes and ovaries within each proglottid suggest the alternative interpretation (**b**)

The new study does not cover gene expression patterns in the growing strobila. Thus, we have no molecular evidence as to the presence and localization of anterior or posterior molecular markers along the chain of proglottids. The iterative organization of the strobila suggests that markers of AP relative position will likely be repeated in each proglottid, as is observed in other species with segmented body patterning. However, given that posterior markers are found in the posterior part of the scolex, how could the proglottids produced caudally from it take on a still more posterior identity, as required if the strobila is really concordant with the scolex in AP polarity? Instead, we can speculate that the tapeworm’s growth zone produces tissues with reversed polarity compared to the scolex (Fig. 3). Three tentative lines of argument can be brought in support of this suggestion.

First, double-head regeneration in planarians suggests that AP polarity is easily reversed in some animals. In these flatworms, free-living relatives of the tapeworms and among the most popular model systems in regeneration studies, AP polarity is established through the Wnt-signalling system that Koziol et al. [8] have used to trace axial polarity in tapeworms. However, in the backward-facing regenerating blastema of planarians deprived of their posterior third, a second ‘head’ is produced from a regenerated region with inverted polarity in case of interruption both of neural signals through the ventral nerve cord and of the continuity of normal gap-junctional communication [9], a scenario that likely applies to the unstructured germinal layer of the metacestode at the time a new scolex starts differentiating from it.

Second, there are examples of asexual reproduction by reverse-polarity budding, such as in the acoel *Convolutriloba retrogemma*. Acoels are not classified nowadays with planarians, or within flatworms, as they were in the past, and thus do not qualify as close relatives of tapeworms. However, they are a sensible choice for comparison here because of their poorly expressed AP polarity, likely comparable to the condition in larval (oncosphere) tapeworms. Some acoels reproduce asexually, each via a peculiar mechanism. In *C. retrogemma*, the animal produces posterior (and posteriorly directed) buds with AP polarity opposite to that of the parent; other species of the same genus reproduce instead by fission whereby the overall AP polarity is conserved throughout the whole process [10].

The occurrence of either process (fission or budding) in members of the same acoel genus suggests that the divide between these two kinds of asexual reproduction is less fundamental than commonly accepted [10]. Perhaps the main difference between fission and budding depends on the position of the proliferative zone relative to the most posterior positional marker in the animal. Cells proliferating in front of this marker will acquire a positional value depending on their position along the animal’s AP axis; thus, any transversal fission plane eventually formed will separate concordantly oriented derivatives (segments or zooids), whereas those produced by a germinal zone behind the posterior marker can only acquire more anterior values, thus behaving as a bud.

Third, the comparative anatomy of the sexual system is somehow at odds with the conventional interpretation of the AP polarity of tapeworms. In the vast majority of cestodes, although not in all of them [3], the testes are anterior to the ovaries. This arrangement is repeated in all proglottids and is also found in the Amphilinidea, Gyrocotyloidea and Caryophyllidea, “monozoic” tapeworms whose body is not articulated into proglottids. The relative position of testes and ovaries is, however, the opposite in most of noncestode flatworms, including the sister group of cestodes, the monogeneans. It is not easy to speculate about a mechanism able to reverse this aspect of body syntax while leaving the animal’s overall polarity unchanged [7, 8].

Future research through which the polarity of the strobila will eventually be fixed will also probably settle the question of the monozoic versus polyzoic nature of tapeworms, that is, whether proglottids are more sensibly comparable to segments of an individual, or to zooids of a colony.
